# Real-World Endoscopic and Histologic Outcomes in Ulcerative Colitis Patients: A Retrospective Cohort Study

**DOI:** 10.3390/biomedicines11071860

**Published:** 2023-06-29

**Authors:** Monica State, Paul Balanescu, Theodor Voiosu, Andreea Bengus, Andrei Voiosu, Andrei Coman, Petronel Mustatea, Lucian Negreanu, Radu Bogdan Mateescu, Cristiana Popp

**Affiliations:** 1 Gastroenterology Department, Colentina Clinical Hospital, 020125 Bucharest, Romania; monicastate4@gmail.com (M.S.); andreeabengus@yahoo.com (A.B.); andreivoiosu@gmail.com (A.V.); bogmateescu@gmail.com (R.B.M.); 2 Internal Medicine Department, Carol Davila University of Medicine and Pharmacy, 050474 Bucharest, Romania; plbalanescu@gmail.com (P.B.); negreanu_99@yahoo.com (L.N.); 3 Pathology Department, Colentina Clinical Hospital, 020125 Bucharest, Romania; andreiicoman08@gmail.com (A.C.); brigaela@yahoo.com (C.P.); 4 Pathology Department, Carol Davila University of Medicine and Pharmacy, 050474 Bucharest, Romania; 5 Surgery Department, Ion Cantacuzino Clinical Hospital, 011437 Bucharest, Romania; petdoc_line@yahoo.com; 6 Gastroenterology Department, Emergency University Hospital, 050098 Bucharest, Romania

**Keywords:** inflammatory bowel disease, ulcerative colitis, mucosal healing, histologic healing

## Abstract

Background: Histologic activity has emerged as an aspirational therapeutic goal in ulcerative colitis management. It is not yet a formal treatment target in ulcerative colitis. However, it could be used as an adjunct to mucosal healing to represent a deeper level of healing. We investigated mucosal and histologic remission rates and potential predictors of these outcomes in a cohort of UC patients. Methods: We conducted a retrospective analysis of data collected from UC patients enrolled in an ongoing prospective cohort study. Mucosal healing was defined as Mayo endoscopic score = 0. Results: A total of 131 patients with ulcerative colitis were enrolled in our study and were prospectively followed for a median length of 2 years (range 0–5 years), totaling 266 study visits. Mucosal healing was recorded for 27 patients at 70 (26%) different study visits. For patients with mucosal healing, histologic remission was achieved in 18/27 (66%) patients. On univariate analysis, sustained clinical remission, SIBDQ scores ≥ 5.5, CRP ≤ 5 mg/dL and absence of corticotherapy were associated with mucosal healing and SIBDQ scores ≥ 5.5 and CRP ≤ 5 mg/dL with histologic healing, respectively. After logistic regression analysis, none of the investigated factors were associated with mucosal and histologic healing. The number of CD8+ intraepithelial lymphocytes (IELs) was significantly greater than the number of CD4+ IELs in periods of disease activity, as well as during mucosal healing (*p* < 0.01 in both cases). Conclusions: Mucosal healing and histologic remission rates are low in real-life settings. The results of univariate analysis indicate that a good quality of life (SIBDQ score) and normal inflammatory markers (CRP) are associated with mucosal and histologic healing. However, frequently used patient- and disease-related factors, including mucosal healing, are not reliable predictors for histologic remission. Greater CD8+ lymphocyte involvement and higher CD8+/CD4+ distribution can have a meaningful impact on understanding the pathogenesis and natural history of ulcerative colitis, as well as future treatment options for lymphocyte-targeting medications.

## 1. Introduction

Ulcerative colitis (UC) is a chronic, relapsing disorder characterized by many knowledge gaps, from its etiology to optimal treatment strategies and monitoring methods [1]. This field has been thoroughly researched in recent decades, with significant progress towards achieving disease control. Its therapy has been traditionally focused on disease-related symptoms; however, most robust outcomes are now proposed by experts and society guidelines. The STRIDE initiative [2], which encompasses evidence- and consensus-based recommendations for treat-to-target strategies, also includes statements regarding histologic outcomes in UC.

Histologic normalization is not yet a formal treatment target in ulcerative colitis. However, it could be used as an adjunct to mucosal healing (MH) to represent a deeper level of healing. There is no validated definition of histological remission in UC. There are numerous different histological scoring systems for UC, but none have been fully validated [3]. One of the most widely used tools for measuring histological activity in UC is the Geboes score. The Geboes Index includes five features (architectural change, lamina propria neutrophils and eosinophils, neutrophils in epithelium, crypt destruction, and erosion or ulceration). This is the best-validated tool, with interobserver variability κ 0.59–0.70 indicating moderate to good agreement between three specialist pathologists [4]. Usually, histologic healing (HH) is defined as the lack of ulceration and activity (intraepithelial neutrophils), the microscopical aspects ranging from the complete normalization of the mucosa to persistent architectural distortion and basal plasmacytosis without signs of activity [5]. A recent ECCO position states that, given the lack of dedicated validated scores in CD, the Geboes Score, Robarts Histopathology Index and Nancy Histological Index could also be used for scoring intestinal biopsies in CD patients [6]. Furthermore, the Geboes score as well as the Nancy index and the RHI can all predict the Mayo endoscopic subscore and fecal calprotectin levels [7]. At the other end, some immunohistochemical features, such as the CD4+ and CD8+ lymph cells, are beginning to raise interest as technical advances facilitate their evaluation [8,9]. CD4+ lymphocytes are the main inflammatory cells involved in ulcerative colitis’ pathology, while CD8+ cells can have pro-inflammatory and immunosuppressant effects. As studies begin to investigate the functional role of intraepithelial lymphocytes in IBD and their therapeutic potential, more consideration must be given to how intraepithelial lymphocytes function in the context of both active disease and remission [10].

Endoscopic MH does not exclude persistent microscopic inflammation, both acute and chronic, which is a harbinger of clinical relapse [11]. As a consequence, histologic activity is becoming more and more important not only for diagnostic purposes but as a prognostic tool, since patients that achieve HH are experiencing better outcomes in all usually evaluated parameters (flares, need for surgery, days of hospitalization, need for therapy escalation, incidence of neoplasia) [2].

The potential impact of setting histologic healing as a treatment target is undeniable and needs further research. Current data for UC patients show that HH is reached in only 15.0% to 44.9%, according to drug class and patient population [1].

We conducted a retrospective predictive cohort study and aimed to determine MH and HH rates. As a secondary outcome measure, we assessed clinical and biological parameters that are associated with histological healing in UC patients. We also investigated the relationship between CD4+ and CD8+ lymphocytes expression and MH.

## 2. Materials and Methods

### 2.1. Patients

IBD patients treated at a tertiary center (Colentina Clinical Hospital, Bucharest, Romania) were enrolled in an ongoing prospective IBD cohort. All patients with a confirmed diagnosis of UC, older than 18 years of age, were invited to participate in this cohort study. The study was conducted in accordance with the Declaration of Helsinki and approved by the local Ethics Committee of Colentina Clinical Hospital. At the index visit, patients were enrolled regardless of their clinical or endoscopic status (remission or flare). Participants were scheduled for evaluation every 12 months, but additional evaluations were performed in cases of worsening clinical condition. The main data collected at each study visit included clinical aspects (disease activity, ongoing medication, smoking status, quality-of-life scores, the Short Inflammatory Bowel Disease Questionnaire (SIBDQ)), inflammatory markers (C-reactive protein) and endoscopic and histological findings, as previously described [12]. Patients with significant comorbidities (i.e., decompensated heart failure or liver cirrhosis, advanced renal failure, significant neurological impairment, etc.), as well as enteral ostomy patients, were excluded from this analysis.

### 2.2. Evaluation of Disease Activity

Clinical disease activity was quantified using the partial Mayo score [13]. Clinical remission was defined as a partial Mayo score < 2. Endoscopic activity was assessed using the Mayo endoscopic score [14]. MH was defined as Mayo endoscopic score of 0. Colonoscopies were performed by four gastroenterologists with experience in IBD and the histology results were reported by an experienced digestive pathologist. The biopsy protocol included two tissue samples from each colonic segment (left colon, right colon, rectum) from the most affected area. In the case of MH, two random samples were collected from the colon. Based on the recommendations of a recent international consensus, a minimum of 2 biopsies/segment or area biopsied is considered appropriate to measure disease activity in UC, and a minimum of 2 biopsies/segment or area biopsied is considered appropriate to measure disease activity in UC [15].

All biopsies from these patients were immediately immersed in 10% buffered formalin, then automatically processed using a Leica ASP300 S Fully Enclosed Tissue Processor and embedded in paraffin. At least six 3 μm sections from two different levels were obtained for usual and special stains. Histologic activity was evaluated using Geboes score (GS). Histological healing is defined as GS ≤ 2.0 (absence of epithelial neutrophils) and histological response is defined as GS < 3.0 [6].

Expression and patterns of distribution of CD4+ and CD8+ T lymphocytes was analyzed by immunohistochemistry (using a fully automatic immunohistochemistry Bond-Max staining system) on biopsies from the MH period and from a previous or a subsequent activation of the disease. Expression was reported as the number of intraepithelial CD4+ and CD8+ cells per 100 colonocytes, counted in a hot spot, while the pattern of distribution was reported as nodular or scattered.

Patients that achieved MH during follow-up (had at least one visit during disease activity followed by a study visit with recorded clinical remission and MH) were included in a subgroup analysis, with a focus on disease and patient characteristics that could predict histologic healing.

### 2.3. Statistical Analysis

All statistical analyses were performed using IBM SPSS version 20.0 for Windows (IBM Corp., Armonk, NY, USA). Statistical analysis of the study data was performed by a biomedical statistician. Data analysis included descriptive statistics computed for continuous variables, expressed as mean and standard deviation (SD). Categorical variables were described as counts and percentages and analyzed by the Chi-Square test. Univariate analysis was used to determine the potential impact of disease- and patient-related factors on selected outcomes (MH). We used nonparametric tests for variables with non-normal distribution (Mann–Whitney U) and Student *t*-test for variables with normal distribution. We also conducted logistic regression, including in the model for all variables of potentially clinical relevance (age, gender, smoking status, treatment, disease extension, disease duration, endoscopic severity) to determine relevant factors for achieving MH and HH. Variables with >10% missing data were excluded from the statistical analysis models. A *p*-value < 0.05 was considered statistically significant.

## 3. Results

### 3.1. Patients

A total of 131 patients were enrolled in our study and were prospectively followed for a median length of 2 years (range 0–5 years), totaling 266 study visits. At enrollment, the majority of patients (96, 73%) had endoscopic activity, with only half of them (73, 55%) having clinical disease activity. For patients in clinical remission, the median duration of remission was 8.5 months. At baseline, corticotherapy was ongoing for 32 (24%) patients, and additionally, 28 (21%) had previous exposure to steroids. Quality of life, as indicated by the SIBDQ, was suboptimal (<5.5 [16]) at enrollment. The baseline characteristics of patients are summarized in Table 1. Smoking status was not recorded in 10/131 (7%) patients.

At baseline, the majority of patients were receiving oral 5-aminosalicylic acids (80/131, 61%), followed by biologics (27/131, 20%) and azathioprine (12/131, 9%). All patients treated with biologics were receiving an anti-TNF agent, infliximab (22/131, 16%) or adalimumab (5/131, 3%). Three patients receiving infliximab were on combination therapy with azathioprine.

### 3.2. Endoscopic Activity

The median value for the endoscopic Mayo score at baseline was 1 (mild activity). The endoscopic inflammatory burden is summarized in Table 1. During the follow-up period, MH was recorded for 27 (20%) patients at 70 (26%) different study visits.

**Figure 1:**
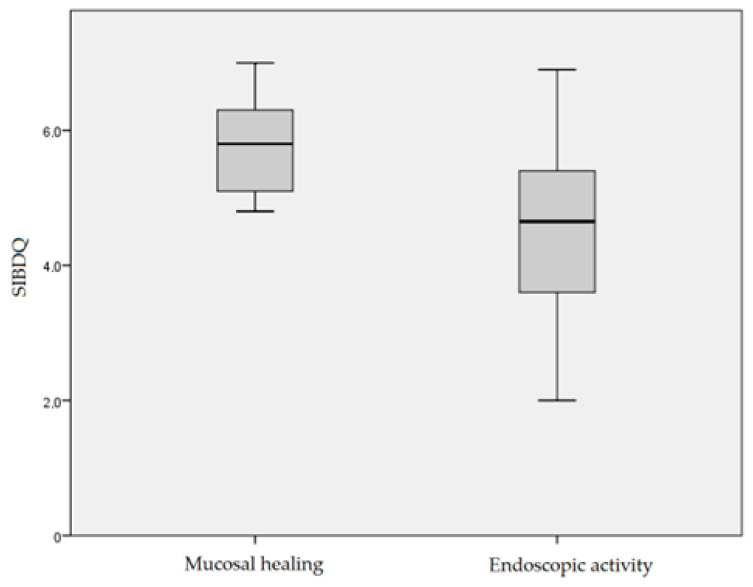
SIBDQ variability according to endoscopic activity.

Presence of endoscopic activity was associated with significantly lower SIBDQ (median 4.8 vs. 5.7 in those with MH *p* < 0.001 Mann–Whitney U) (Figure 1), increased inflammatory burden (median CRP levels 1.61 mg/L vs. 4.05 mg/L, *p* < 0.001 Mann–Whitney U) and biologics use (*p* < 0.001, Fisher’s exact test).

On univariate analysis, sustained clinical remission (>12 months [17]), SIBDQ ≥ 5.5, CRP ≤ 5 mg/dL and absence of ongoing corticotherapy were associated with MH at follow-up (Table 2).

On logistic regression, none of the factors of potentially clinical relevance (age, gender, smoking status, corticosteroids and biologics, disease extension, disease duration and endoscopic severity) were associated with MH (Table 3).

### 3.3. Histologic Healing

From the total of 70 visits, histopathology results were available for 48 of them. In 18/48 (37%) study visits with no inflammatory activity at endoscopy (MH), biopsies were indicative of histologic remission. On univariate analysis, SIBDQ scores ≥ 5.5 and normal CRP levels were associated with histologic healing (Table 4).

After logistic regression analysis, none of the investigated factors (clinical activity, disease duration, age, gender, diagnostic, smoking, corticosteroid or biologics use), including endoscopic activity, was associated with histologic remission.

We identified 20 patients with UC that achieved MH during follow-up (Table 5). In addition to the 28/131 who already had MH at the time of enrollment, 27/131 (20%) had only clinical remission, partial clinical response or uncontrolled disease (56/131, 42%).

**Figure 2:**
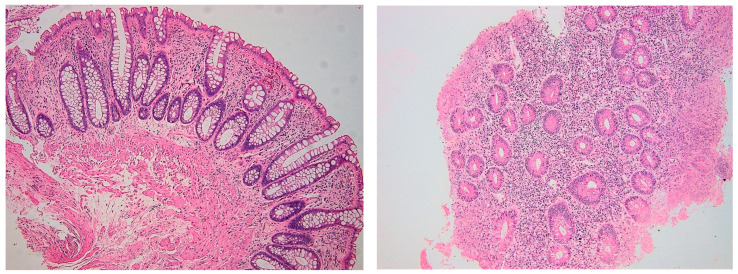
Mucosal healing (**left**) and active disease (**right**) in a patient with UC. In the left image (Geboes score 1.1), there is mild but unequivocally increased chronic inflammatory infiltration without epitheliotropism and without neutrophils. Also, there is a slightly modified plasma cell gradient. In the right image (Geboes score 4.2), note marked inflammatory infiltration with numerous neutrophils, epitheliotropism and crypt destruction. Hematoxylin-eosin, 100×.

For patients that achieved MH, we performed a comparative analysis of histologic characteristics (Geboes score and CD8+ and CD4+ intraepithelial lymphocytes) from baseline (present endoscopic activity) and follow-up (MH) evaluations (Table 6). In biopsies taken during endoscopic MH, histologic activity was variable, but usually inflammatory changes were low-grade. An example of the histologic activity assessment is shown in Figure 2.

**Figure 3:**
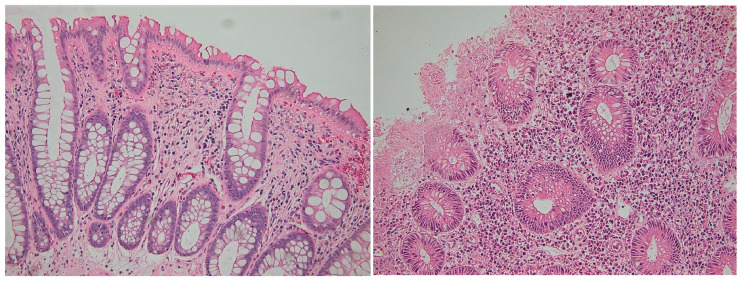
Details of images shown in Figure 2: Mucosal healing (**left**) and active disease (**right**) in a patient with UC. In the left image (Geboes score 1.1), note the chronic inflammation limited in lamina propria, without intraepithelial inflammatory cells. In the right image (Geboes score 4.2), note marked inflammatory infiltration, with numerous neutrophils, epitheliotropism and exulceration. Hematoxylin-eosin, 200×.

**Figure 4:**
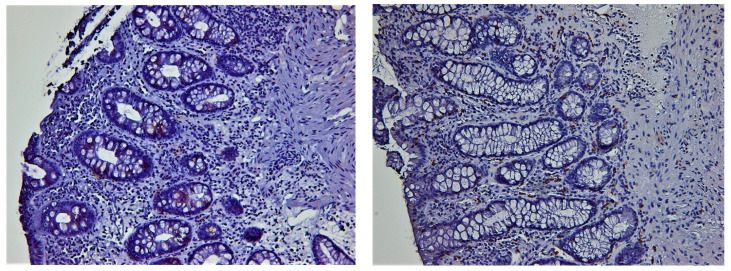
Immunostaining for CD4 (**left**) and CD8 (**right**) in the same case of inactive chronic colitis (endoscopic healing). Note small groups of 2–4 CD4+ lymph cells in cryptic epithelium. On the other hand, intraepithelial CD8+ cells are more frequent but scattered (CD4 and CD8 stain, 200×).

Nine patients had a Geboes score between 0.3 and 1.3 (equivalent to histologic healing), while another eight patients had a Geboes score ≤ 3.1 (without histological activity) [18,19]. During the MH period, only 1 patient had a Geboes score higher than 4.1, while during disease activity 14 were found to have scores higher than 4.1. The number of CD8+ intraepithelial lymphocytes was significantly greater than the number of CD4+ IELs in periods of disease activity, as well as during MH (*p* < 0.01 in both cases) (Table 6). In regard to patterns of distribution, CD8+ cells had a scattered pattern in both intervals, while CD4+ cells had a predominantly nodular distribution in MH while being scattered during periods of disease activity (Figure 3 and Figure 4).

## 4. Discussion

Inflammatory bowel disease research focused on disease course and long-term patient outcomes has produced enough evidence to change management strategies [2]. Treatment is no longer aiming purely at controlling symptoms, but at achieving targets, such as MH, that could translate into effective and sustained disease control (treat-to-target approach). The STRIDE initiative [20], which encompasses evidence- and consensus-based recommendations for treat-to-target strategies, also includes statements regarding histologic outcomes in IBD. However, clinical trials rarely focused on histological activity in IBD beyond its diagnostic role. Available data refer to the histologic response at best, and to various therapeutic agents, mostly biological therapy [21,22,23].

The results of our study show that MH and histologic remission rates are low in real-life settings. In our analysis, only 47/131 (35%) patients had or achieved MH during follow-up and histologic healing was reported for even fewer (18/131, 13%). The literature data show that microscopic inflammation persists in 16–100% of cases of endoscopically quiescent disease [7]. Considering persistent histologic activity has been associated with frequent flare-ups [24], hospitalization [25] and risk of colorectal neoplasia [26], and that endoscopic absence of disease is not a reliable indicator of histologic normalization, focusing on histologic results might become a priority in the near future.

Recent studies suggest that histologic healing might be associated with better long-term clinical outcomes. For example, a retrospective analysis showed that histologic healing was associated with decreased risk of clinical relapse (hazard ratio [HR], 2.05; 95% CI, 1.07–3.94; *p* = 0.031), medication escalation (HR, 2.17; 95% CI, 1.2–3.96; *p* = 0.011) and corticosteroid use (HR, 2.44; 95% CI, 1.17–5.09; *p* = 0.018) [27]. Furthermore, a systematic analysis showed that less-severe clinical disease activity and corticosteroid use were associated with higher histologic remission rates. Similarly, mild clinical disease activity was associated with higher histologic remission rates [28].

Clinical remission, clinical response and endoscopic response are ranked the most important short-term goals in UC. Long-term outcomes include the normalization of inflammatory markers (C-reactive protein, calprotectin), quality of life and histologic healing. However, HH is not yet a formal treatment target in either CD or UC [1].

The investigation of patient- and disease-related factors that correlate with endoscopic and histologic activity could help improve the monitoring of patients and improve disease control. Several noninvasive biomarkers have shown good performance in predicting endoscopic activity [29]. For example, fecal calprotectin has been incorporated into society guideline recommendations and has a clear role in current real-life clinical practice, as it can substitute endoscopic evaluation in certain clinical scenarios. Given the increasing importance of histologic healing, studies have recently begun to investigate the performance of various parameters in predicting this endpoint. For example, the study conducted by Gubatan et al. [30] showed promising results regarding the accuracy of serum cytokines in assessing histologic healing.

In our analysis, commonly used tools such as CRP and quality of life scores (SIBDQ) were indicators of both MH and HH. However, statistical significance was limited to the univariate analysis. Future studies could investigate, in a prospective setting on a greater numbers of patients, the role of noninvasive markers in assessing histologic healing.

We also investigated the relationship between CD4+ and CD8+ lymphocyte expression and MH, with some interesting results. The number of CD8+ intraepithelial lymphocytes was significantly greater than the number of CD4+ IELs in periods of disease activity, as well as during MH (*p* < 0.01 in both cases). More relevant, CD8+ cells had a scattered pattern in both intervals, while CD4+ cells had a predominantly nodular distribution in MH, while being scattered during periods of disease activity. These results set the premise for future studies that could incorporate baseline histologic features for predicting patient outcomes and, more importantly, develop new drugs.

The main limitation of our analysis refers to the small study population and low number of patients with histologic healing (18/131, 13%). The study population was heterogenous, starting from enrollment (patients with both active and inactive disease), disease characteristics, treatment regimens and outcomes. Biopsies were taken in only 48/70 study visits and, as a consequence the HH rate, could be underestimated. Prospectively collected data following a protocol that included a large quantity of data for each patient made statistical analysis possible and generated interesting results. Our limited study population generated interesting results regarding key treatment endpoints in real-life settings. However, the low number of patients limits the generalization of the results.

## 5. Conclusions

The most important conclusion of our study is that MH and histologic healing rates are low in real-life settings. The results of the univariate analysis indicate that good quality of life (SIBDQ score) and normal inflammatory markers (CRP) are associated with MH and HH. However, on logistic regression, frequently used patient- and disease-related factors including MH are not reliable predictors for endoscopic and histologic remission.

## Figures and Tables

**Table 1:** Patient baseline characteristics.

	Ulcerative Colitis
Gender, male/female, n (%)	83 (63)/48 (37)
Age, mean (SD), years	43 (14)
Smoker/non-smoker, n (%)	16 (12)/105 (80)
Disease extension, n (%)	E1 29 (22), E2 34 (26), E3 58 (44)
Disease duration, mean (SD), years	5.0 (6.2)
Clinical activity, n (%)	75 (57)
Active disease at endoscopy, n (%)	100 (76)
Mayo endoscopic score 0/1/2/3, n (%)	23 (17)/43 (32)/29 (22)/26 (19)
Mayo endoscopic score, median	1 (0–3)
SIBDQ, median (min, max)	4.9 (2.0–7.0)
CRP, median (mg/dL)	3.55
Ongoing biologics, n (%)	27 (20%)
Previous exposure to biologics, n (%)	5 (0.3%)
Ongoing corticosteroids, n (%)	32 (24%)

SD = standard deviation, E1 = proctitis, E2 = left colitis, E3 = pancolitis, SIBDQ = the Short Inflammatory Bowel Disease Questionnaire, CRP = C-reactive protein.

**Table 2:** Impact of clinical and biological factors on mucosal healing.

Parameter	Mucosal Healing	Endoscopic Activity	*p* Value
Gender (M/F)	18/65	12/36	0.32
Sustained clinical remission (yes/no)	24/4	27/70	<0.001 *
Ongoing corticosteroids (yes/no)	2/26	30/68	0.004 *
Ongoing biologics (yes/no)	7/21	22/76	0.08
Smoking status (yes/no)	22/3	81/12	0.90
SIBDQ < 5.5 (yes/no)	11/16	54/22	0.01 *
CRP > 5 mg/dL (yes/no)	4/48	40/22	0.01 *

* *p* < 0.05.

**Table 3:** Logistic regression model investigating the role of clinical and biological factors in predicting MH.

Variable	Unstandardized Coefficients		Standardized Coefficients	*t*	*p*
	B	Std. Error	Beta		
Age	0.003	0.006	0.061	0.404	0.688
Gender	−0.086	0.133	−0.095	−0.647	0.521
Disease extension	0.079	0.085	0.154	0.936	0.355
Corticotherapy	0.005	0.080	0.011	0.062	0.951
Biologics	0.020	0.142	0.024	0.138	0.891
Smoking status	−0.191	0.213	−0.134	−0.895	0.376
Disease duration	−0.016	0.010	−0.248	−1.660	0.104

**Table 4:** Univariate analysis model investigating role of clinical and biological factors in predicting HH.

Parameter	Histologic Healing	Histologic Activity	*p* Value
Gender (M/F)	14/6	16/12	0.36
Endoscopic activity (yes/no)	1/17	0/26	0.40
Ongoing corticosteroids (yes/no)	1/11	1/14	0.82
Ongoing biologics (yes/no)	7/10	9/16	0.91
Smoking status (yes/no)	2/16	3/23	0.96
SIBDQ < 5.5 (yes/no)	6/13	15/12	0.01 *
CRP > 5 mg/dL (yes/no)	0/13	8/18	0.05 *

* *p* < 0.05.

**Table 5:** Baseline characteristics for patients that achieved histologic healing during follow-up.

Variable	Ulcerative Colitis with MH at Follow-Up
Gender, male/female, n	9/11
Age, mean (SD), years	49 (15)
Smoker/non-smoker, n	4/15
Disease extension, E1/E2/E3, n	4/10/6
Disease duration, mean (SD), years	5.1 (8.3)
Corticosteroids, n	0
Biologics, n	12
Partial Mayo score, median	1.5
Endoscopic Mayo score, median	1
SIBDQ, median (min max)	5.3 (2.8, 6.8)
CRP, median (mg/dL)	5.86

**Table 6:** Histologic assessment at baseline and follow-up visits for patients that achieved MH.

Parameter	Endoscopic Activity	Mucosal Healing	*p* Value
Geboes score, median (min–max)	4.3 (1–5)	2.1 (0–5)	<0.01
CD4+ IELs, median (min–max)	6 (0–25)	4 (0–13)	*p* < 0.01
CD8+ IELs, Median (min–max)	16 (5–42)	10.5 (0–26)	*p* < 0.01

## Data Availability

The data presented in this study are available on request from the corresponding author. The data are not publicly available due to ethical considerations.
